# The clinical trial landscape of osteosarcoma: integrating trial data, immunotherapeutic trends, and biomarker insights

**DOI:** 10.3389/fimmu.2026.1790067

**Published:** 2026-03-10

**Authors:** Lifeng Ge, Tianhao Xu, Xiaodong Gu, Xuekang Pan, Tian Gao, Huigen Lu

**Affiliations:** 1 Department of Orthopaedics, The Second Affiliated Hospital of Jiaxing University, Jiaxing, China; 2 Department of Oncology, The Second Affiliated Hospital of Jiaxing University, Jiaxing, China

**Keywords:** alkylating agents, anthracyclines, anti-angiogenic agents, antimetabolites, apatinib, CD8a, conventional chemotherapy, CTLA-4 blockers

## Abstract

Osteosarcoma, the most aggressive primary malignant bone tumor, has stagnant therapeutic outcomes despite decades of standard MAP chemotherapy and surgery; 5-year overall survival (OS) is <30% for metastatic/recurrent cases. Plagued by genomic heterogeneity, immunosuppressive TME, and low immunogenicity, emerging immunotherapies lack robust large-scale clinical validation. We systematically analyzed 864 interventional osteosarcoma trials from Trialtrove (as of September 2025). Results showed trial numbers peaked at 54 in 2021, with 77.3% past (completed/terminated) and over 94% in phase I/II (only 3.6% phase III-IV). Geographically, the U.S. dominated (60.9%, focusing on immunotherapy/targeted therapy), while low- and middle-income countries (LMICs) accounted for <2% of trials despite bearing 40% of the global disease burden. Conventional chemotherapy remains the cornerstone, with immuno-oncology (540 trials) as the leading novel strategy; top targets include VEGFR2 (104), PD-1 (70), and mTOR (60). Biomarker use was imbalanced: liver/nutritional markers prevailed, while key immune/genomic biomarkers (CD8A, TP53) were underrepresented (<8% combined). Key challenges include severe trial-phase imbalance, global disparities, and preclinical-clinical gaps; opportunities lie in synergistic novel therapies (ICI combinations, GD2-targeted CAR-T) and decentralized clinical trials (DCT). Future priorities: accelerate late-phase trials for promising regimens, reduce global disparities via regional consortia, integrate precision biomarkers for patient stratification, and translate TME insights into trials. This analysis highlights the need to shift from conventional chemotherapy optimization to precision-driven, globally equitable strategies to improve outcomes for high-risk osteosarcoma patients.

## Introduction

Osteosarcoma, the most aggressive primary malignant bone tumor, exhibits a bimodal age distribution—primarily affecting adolescents/young adults (AYAs, 10–24 years of age) and adults >65 years of age—and accounts for approximately 3% of pediatric cancers and 1% of adult solid malignancies worldwide (1, 2). Annually, it affects 3–5 individuals per million, with over 80% of tumors arising in the metaphyses of long bones (e.g., distal femur, proximal tibia) (3). Despite decades of reliance on the standard frontline regimen—neoadjuvant MAP (high-dose methotrexate, doxorubicin, cisplatin) chemotherapy plus surgical resection—therapeutic outcomes remain stagnant: 5-year overall survival (OS) reaches 60–70% for localized disease but drops to <30% for metastatic, recurrent, or refractory cases (4, 5). Notably, patients with relapsed unresectable osteosarcoma have a 4-month event-free survival (EFS) rate of only 12%, which underscores a critical unmet clinical need (6).

The therapeutic challenges of osteosarcoma stem from three key biological characteristics: (1) profound genomic heterogeneity, including TP53 mutations in >90% of cases, RB1 deletions in 30% of cases, and frequent chromothripsis (7, 8); (2) an immunosuppressive tumor microenvironment (TME) populated by M2-polarized tumor-associated macrophages (TAMs), myeloid-derived suppressor cells (MDSCs), and PD-L1-expressing tumor cells that impair T cell-mediated antitumor responses (9, 10); (3) low inherent immunogenicity, which limits the efficacy of single-agent therapies (11). While emerging strategies—such as immune checkpoint inhibitors (ICIs), chimeric antigen receptor (CAR)-T cells targeting GD2/B7-H3/HER2, and γδ T cell adoptive transfer—show preclinical promise, they lack robust validation in large randomized trials (2, 12).

To address this gap, we systematically analyzed 864 interventional osteosarcoma trials from the Trialtrove database (as of September 2025), integrating data on trial status, geographic distribution, therapeutic focus, and biomarker application. Our objective was to characterize the current research landscape, identify unmet needs, and guide stakeholder actions—with key findings presented herein.

## Key trial landscape findings

**Figure 1:**
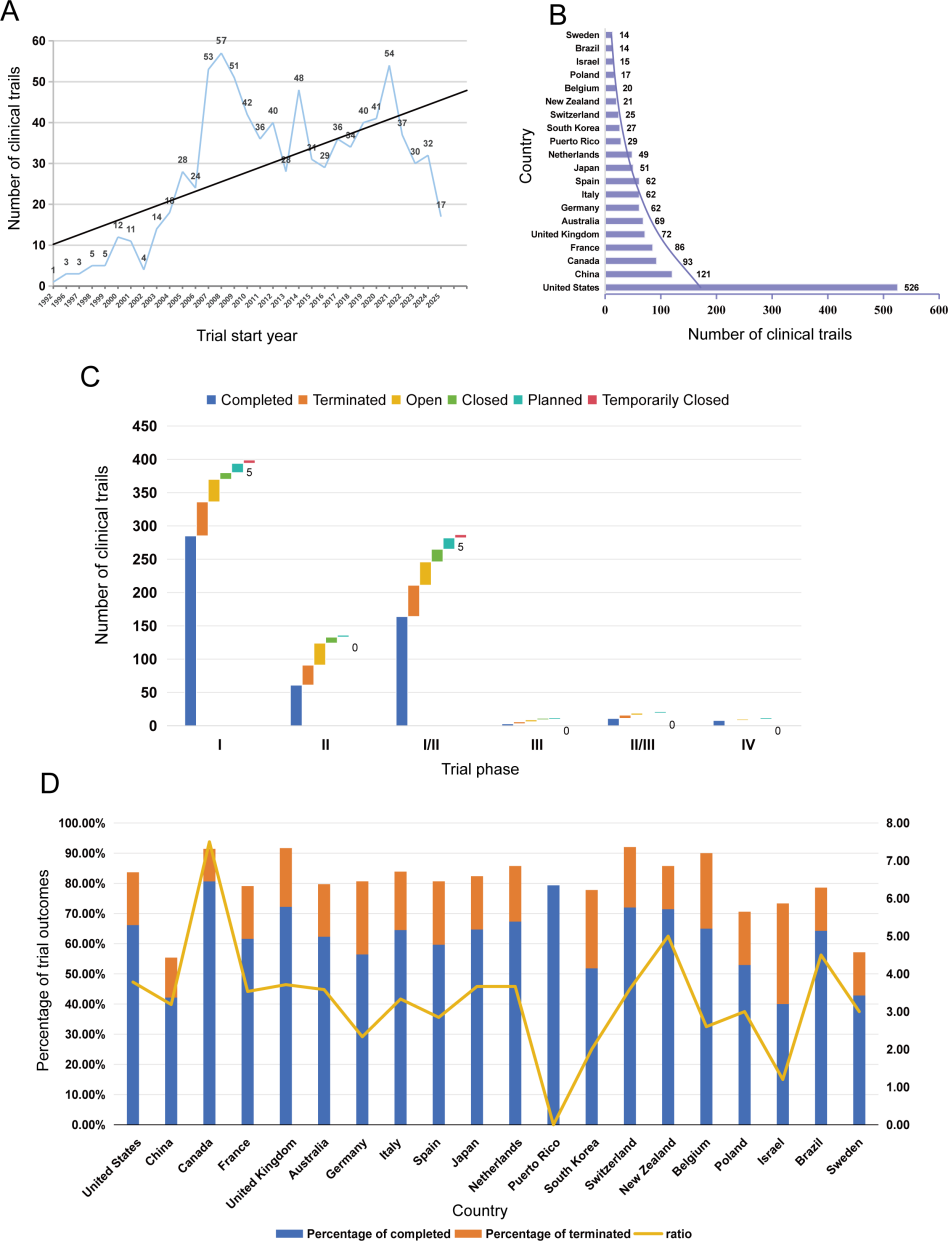
Trends and distribution of osteosarcoma clinical trials. **(A)** Temporal trends by trial start year (1992–2025); **(B)** Geographic distribution (top 20 countries); **(C)** Distribution by trial phase and status. **(D)** Proportional distribution of trial outcomes and completion-to-termination ratios across top countries.

A total of 864 eligible interventional trials were included. Temporal trends revealed steady growth (**Figure 1A**), from 1 trial in 1992 to a peak of 54 trials in 2021, followed by a slight decline during 2022–2025. Most trials (77.3%) were categorized as “past” (i.e., 532 completed and 136 terminated); 18.4% were ongoing, and 4.3% were planned. Trial termination was primarily driven by insufficient recruitment, business decisions, and lack of efficacy, with safety issues accounting for only 4.4%.

Trial phases were heavily skewed toward early stages (**Figure 1C**): phase I (46.2%), phase II (33.2%), and combined phase I/II (15.7%) trials collectively constituted >94%, while late-phase trials (phase III–IV) accounted for only 3.6%. Each trial phase is characterized by distinct core research objectives, evidence generation value and study designs that define their roles in clinical translation (**Supplementary Table 1** for details).This analysis reveals that trial termination risk is not uniformly distributed across phases (**Supplementary Figure 2**): early-phase trials (phase II) and late-phase confirmatory trials (phase III) are disproportionately affected by termination, with phase II showing the highest absolute termination rate among early phases and phase III exhibiting an equal likelihood of termination and completion. In contrast, phase I and phase IV trials demonstrate the highest completion rates, with phase IV showing no terminated trials at all. This pattern suggests that therapeutic development in osteosarcoma faces critical bottlenecks at two key translational junctures: the transition from exploratory efficacy testing (phase II) to confirmatory validation (phase III), and the execution of large-scale confirmatory trials (phase III) themselves, where failure rates are alarmingly high.

This extreme phase distribution reflects profound challenges in advancing therapies to large-scale validation for this rare malignancy, and more critically, constitutes a pivotal barrier to clinical translation. The overwhelming predominance of early-phase trials means that the vast majority of osteosarcoma therapeutic strategies only have preliminary safety data and exploratory efficacy findings, lacking the high-quality confirmatory evidence from large-sample RCTs that is indispensable for translating novel therapies from preclinical research and early-phase exploration into routine clinical practice. The high termination rates in phase II and phase III further exacerbate this translational gap, as promising therapeutic signals from early exploration often fail to progress to definitive validation, limiting the availability of evidence-based treatment options for patients with osteosarcoma.

Geographically, the top 20 countries accounted for 95% of trials, with substantial disparities (**Figure 1B**). The United States (U.S.) led with 60.9% of trials, with a strong focus on immunotherapeutics and targeted therapy; China followed with 14.0%, where 23.1% (within specific subgroups.)of trials focused on CAR-T cells. Western European countries (France, United Kingdom [UK], Germany, Italy, Spain) each contributed 60 to 86 trials, while low- and middle-income countries (LMICs, e.g., Brazil) accounted for <2% of global trials. Notably, LMICs conducted almost no trials evaluating complex immunotherapies (e.g., CAR-T cells, ICI combinations)—despite carrying 40% of the global osteosarcoma burden (12).

To address whether trial volume correlates with favorable outcomes, we analyzed country-level trial completion rates, termination rates, and completion-to-termination ratios (**Figure 1D**).

This analysis reveals that trial volume does not directly equate to superior outcomes: the U.S., while leading in trial numbers, only achieved moderate completion and termination rates. Conversely, smaller-volume countries like Puerto Rico and Canada achieved the most favorable outcomes, with Puerto Rico showing no terminated trials at all. LMICs such as Brazil maintained reasonable completion-to-termination ratios (~4.5), despite their limited trial participation. These findings highlight that beyond sheer trial numbers, regional research infrastructure, patient recruitment strategies, and trial design rigor are critical determinants of trial success in osteosarcoma therapeutic development.

**Figure 2:**
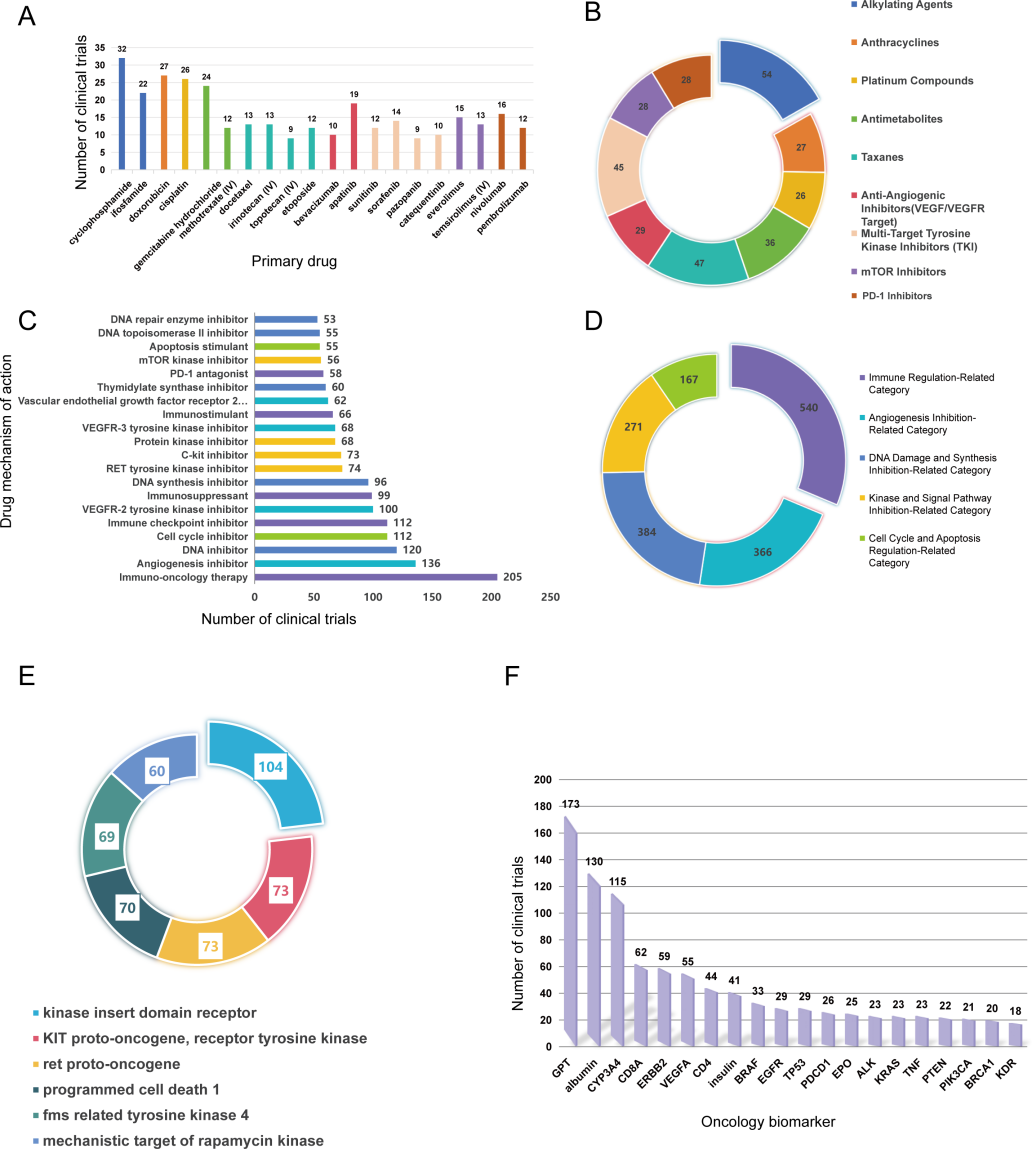
Therapeutic focus and biomarker landscape of osteosarcoma trials. **(A)** Number of trials by primary drug under investigation (top 20); **(B)** Types of primary drugs under investigation; **(C)** Number of trials by primary drug mechanism of action (top 20); **(D)** Classification of primary drug mechanisms of action; **(E)** Drug targets investigated in trials; **(F)** Number of trials by oncology biomarker.

In the landscape of osteosarcoma therapeutics, treatment strategies range from long-established conventional chemotherapy to emerging immunotherapies and targeted agents—each with distinct clinical roles and unresolved challenges (**Figures 2A, B**). Conventional cytotoxic chemotherapy remains the cornerstone of care, and clinical trials predominantly focus on four drug classes: alkylating agents (e.g., cyclophosphamide, ifosfamide), anthracyclines (doxorubicin), platinum compounds (cisplatin), and antimetabolites (gemcitabine). These agents are rooted in decades-old therapeutic regimens, which starkly reflects the lack of breakthroughs in first-line treatment for localized osteosarcoma.

Meanwhile, emerging immunotherapies and targeted agents are gaining research attention but remain underdeveloped. Immune checkpoint inhibitors (ICIs)—notably nivolumab and pembrolizumab—are under evaluation, yet their trial count lags significantly behind that of chemotherapy (**Figures 2A, B**). Their modest efficacy as monotherapies (objective response rate [ORR] of 4.5–6.7%) further underscores the urgency of exploring combination strategies, such as regimens combining PD-1 inhibitors and CTLA-4 blockers. Anti-angiogenic agents (apatinib, sorafenib, sunitinib) target vascular endothelial growth factor receptor 2 (VEGFR2) to disrupt tumor angiogenesis, demonstrating moderate activity (4-month progression-free survival [PFS] of 33–46%) but facing inherent limitations due to intrinsic or acquired drug resistance. Additionally, mTOR inhibitors (everolimus, temsirolimus)—which regulate cell growth and metabolism by targeting the mechanistic target of rapamycin (mTOR)—are tested exclusively in small-scale trials, often in combination with chemotherapy; their precise therapeutic role in osteosarcoma remains undefined.

The most studied drug targets in osteosarcoma research align with addressing its core biological hallmarks, focusing on three key categories: kinases, immune checkpoints, and metabolic regulators (**Figure 2E**). Kinases dominate this landscape: VEGFR2 (also known as KDR) is the top target, investigated in 104 trials—driven primarily by the development of anti-angiogenic agents. Multi-kinase inhibitors (e.g., sorafenib) also target KIT and RET proto-oncogenes, though their direct relevance to osteosarcoma pathogenesis remains incompletely validated. Immune checkpoints, particularly programmed cell death 1 (PD-1/PDCD1), are targeted by ICIs in 70 trials, reflecting efforts to reverse T-cell exhaustion within the immunosuppressive TME. Additionally, the mechanistic target of rapamycin (mTOR) is the focus of 60 trials (involving everolimus and temsirolimus), with the goal of disrupting abnormal cell proliferation and metabolic reprogramming in osteosarcoma.

The mechanisms of action (MOA) of osteosarcoma therapeutics reveal a dual pattern: persistent reliance on traditional cytotoxic strategies and active exploration of novel immunomodulatory approaches (**Figures 2C, D**). Immuno-oncology therapy is the largest category, with 540 trials encompassing ICIs, cancer vaccines, and adoptive cell therapy—reflecting growing interest in overcoming osteosarcoma’s immunosuppressive TME (characterized by PD-L1 expression and M2-polarized TAMs). Anti-angiogenesis, evaluated in 366 trials, targets tumor vascularization via VEGFR2 inhibition to deprive tumors of nutrients; however, long-term efficacy is constrained by resistance mechanisms such as vessel co-option.

Traditional cytotoxic mechanisms remain critical for localized disease: DNA inhibition (384 trials) and cell cycle inhibition (167 trials) rely on alkylating agents and platinum compounds to induce DNA damage or arrest cell division, respectively (**Figure 2D**). Nevertheless, these approaches fail in metastatic or recurrent settings due to acquired drug resistance. Finally, immune checkpoint inhibition (112 trials)—a subset of immuno-oncology—focuses on blocking PD-1/PD-L1 or CTLA-4 pathways to reinvigorate T-cell-mediated antitumor responses (**Figure 2C**), representing a promising but still evolving strategy.

To address whether trial outcomes differ across therapeutic classes, we analyzed completion and termination rates for major MOA categories (**Supplementary Figure 2**). Immune regulation–related therapies, including ICIs, vaccines, and adoptive cell therapy, displayed the lowest completion rate (46.11%) with a termination rate of 17.22%, driven by both biological futility in the immunosuppressive TME and feasibility constraints such as slow recruitment and cell therapy manufacturing. By comparison, angiogenesis inhibition exhibited a high completion rate (68.03%) and low termination rate (9.29%). DNA damage inhibition showed a completion rate of 56.77% and termination rate of 22.14%, mostly due to acquired resistance in advanced disease. Kinase inhibition achieved the highest completion rate (76.01%) and lowest termination rate (7.75%), supported by well-defined targets and robust preclinical evidence. Cell cycle and apoptosis regulation yielded a high completion rate (74.25%) with termination related to toxicity or limited efficacy in refractory cases(All proportions in this paragraph represent percentages within specific subgroups).

This analysis indicates that immunotherapy trials face a dual burden: while feasibility issues contribute to trial termination, the low completion rate is driven mainly by biological barriers, particularly the immunosuppressive TME of osteoscoma that attenuates T cell–mediated anti-tumor activity. In contrast, targeted and conventional cytotoxic therapies achieve substantially higher completion rates owing to established mechanisms and clinical paradigms. These observations highlight that future immunotherapy trials will require optimized design (e.g., adaptive dosing, biomarker stratification) and TME-modulating strategies to surmount both biological and translational obstacles.

Biomarker utilization was imbalanced: liver function (glutamic-pyruvic transaminase [GPT], 20.0%) and nutritional status (albumin, 15.1%) markers dominated (**Figure 2F**), while immune-related (CD8A, PDCD1) and genomic (TP53, BRCA1) biomarkers—critical for precision immunotherapy—were underrepresented (accounting for <8% combined). Most biomarkers were used for inclusion/exclusion criteria (e.g., GPT in 82.2% of trials, CYP3A4 in 86.3% of exclusion criteria), with fewer integrated into efficacy endpoints (e.g., CD8A in 40.3%) or result analysis (e.g., TP53 in 52.8%).

## Conclusion

Our analysis of 864 osteosarcoma clinical trials demonstrates that while the field is limited by key barriers, it holds significant transformative potential—with clear pathways to address the unmet needs of high-risk patients.

Core challenges impeding progress include: (1) A severe trial-phase imbalance: over 94% of trials focus on phase I/II safety evaluation, while phase III–IV confirmatory trials account for only 3.6%. This imbalance is driven by osteosarcoma’s rarity and high trial termination rates (largely due to poor recruitment); (2) Disparities in access and precision: the U.S. leads with 60.9% of trials, while LMICs—bearing 40% of the global burden—contribute <2%; biomarkers are skewed toward safety/metabolic markers, with immune (CD8A, PD-1) and genomic (TP53) markers used in <8% of cases; (3) Gaps between preclinical and clinical research: promising preclinical findings (e.g., CSF-1R inhibitors reversing M2-TAM-mediated immunosuppression) are rarely translated to clinical trials.

Notably, several opportunities exist to advance the field: (1) Emerging therapies (ICI combinations, GD2-targeted CAR-T cells, anti-angiogenics such as cabozantinib) show synergistic potential, offering alternatives to stagnant chemotherapy regimens; (2) Methodological advances: decentralized clinical trials (DCTs) using telemedicine and wearables facilitate recruitment, while multi-omics biomarkers improve patient selection for immunotherapies.

Future priorities should include: (1) Accelerating late-phase trials for high-potential combinations (e.g., ICI + anti-angiogenics, optimized CAR-T cell therapies) to confirm efficacy; (2) Reducing global disparities through regional consortia that share resources (e.g., CAR-T cell manufacturing, biomarker testing) with LMICs; (3) Integrating precision biomarkers (PD-L1, CD8A, TP53) for patient stratification; (4) Translating TME insights (e.g., CSF-1R-mediated M2-TAM reprogramming) into mechanistic clinical trials.

In conclusion, osteosarcoma research must move beyond incremental optimization of conventional chemotherapy to adopt precision-driven, globally equitable strategies. Resolving trial-phase imbalances, leveraging emerging therapies, and uniting global stakeholders are critical to improving outcomes for high-risk patients and narrowing the gap between scientific potential and clinical impact.

## Data Availability

The original contributions presented in the study are included in the article/**Supplementary Material**. Further inquiries can be directed to the corresponding author/s.
