# Supplementary material for: The clinical trial landscape of osteosarcoma: integrating trial data, immunotherapeutic trends, and biomarker insights
Source: Front Immunol. 2026 Mar 10;17:1790067. doi: 10.3389/fimmu.2026.1790067 (PMC13008900; doi:10.3389/fimmu.2026.1790067)
Supplement: Supplementary file 2 [file Table2.docx]

**Table**

**Table S1 Summary Table of the Core Significance of Each Phase of Osteosarcoma Clinical Trials**

| **Trial Phase Type** | **Specific Phase** | **Core Evidence** | **Core Objectives/Design** | **Evidence Level/Value** | **Remarks** |
| --- | --- | --- | --- | --- | --- |
| **Phase I/II (Exploratory Stage)** | **Phase I** | **Preliminary safety and tolerability** | **Determine MTD, RP2D; evaluate PK/PD; observe acute adverse reactions** | **Lowest; safety only, no confirmed efficacy** | **-** |
|  | **Phase II** | **Preliminary efficacy + expanded safety** | **Evaluate ORR/CBR/PFS to determine further development** | **Moderate; potential efficacy, not for routine use** | **-** |
|  | **Combined Phase I/II** | **Integrated early safety + preliminary efficacy** | **Synchronous exploration of safety and preliminary efficacy** | **Moderate; exploratory, no confirmatory value** | **Most common for osteosarcoma; shortens R&D, saves samples** |
| **Phase III~IV (Confirmatory/Real-World Stage)** | **Phase III ("Gold Standard")** | **Confirmatory efficacy and benefit-risk** | **Large-sample, multi-center RCT; primary endpoints: OS/EFS** | **Highest; supports approval, guidelines, standard treatment** | **Decisive step to routine clinical use** |
|  | **Phase IV (Post-Marketing)** | **Long-term safety + real-world effectiveness** | **Evaluate long-term toxicity, special population benefits, real-world effect** | **High; optimizes risk-benefit, individualized medication** | **Supplementary for rational post-marketing use** |

Figures





Figure S1 Proportional Distribution of Trial Outcomes Across Clinical Phases in Osteosarcoma Trials





Figure S2 Proportional Distribution of Completed and Terminated Trials Across Major Mechanism-of-Action (MOA) Categories in Osteosarcoma Therapeutic Development
